# Menstrual blood-derived mesenchymal stem cells combined with collagen I gel as a regenerative therapeutic strategy for degenerated disc after discectomy in rats

**DOI:** 10.1186/s13287-024-03680-w

**Published:** 2024-03-13

**Authors:** Li Yu, Honghao Wu, Shumei Zeng, Xiaojian Hu, Yuxu Wu, Jinhong Zhou, Li Yuan, Qingqing Zhang, Charlie Xiang, Zhiyun Feng

**Affiliations:** 1 Department of Operating room, The First Affiliated Hospital, Zhejiang University School of Medicine, Hangzhou, 310003 China; 2 Department of Orthopedic Surgery, The First Affiliated Hospital, Zhejiang University School of Medicine, Hangzhou, 310003 China; 3 Department of gynaecology, Zhejiang Hospital, Zhejiang University School of Medicine, Hangzhou, China; 4 Innovative Precision Medicine (IPM) Group, Hangzhou, Zhejiang China; 5 State Key Laboratory for Diagnosis and Treatment of Infectious Diseases, Collaborative Innovation Center for Diagnosis and Treatment of Infectious Diseases, The First Affiliated Hospital, College of Medicine, National Clinical Research Center for Infectious Diseases, Zhejiang University, Hangzhou, Zhejiang China; 6 Building 8-2, 58#, Chengzhan Road, Hangzhou, 310003 China; 7 State Key Laboratory for Diagnosis and Treatment of Infectious Diseases, The First Affiliated Hospital, College of Medicine, Zhejiang University, Hangzhou, China

**Keywords:** Discectomy, Menstrual blood-derived mesenchymal stem cells, Disc repairment, Disc degeneration, Annulus fibrosis defects, Tissue engineering, Paracrine, Growth factors

## Abstract

**Background:**

Annulus fibrosis (AF) defects have been identified as the primary cause of disc herniation relapse and subsequent disc degeneration following discectomy. Stem cell-based tissue engineering offers a promising approach for structural repair. Menstrual blood-derived mesenchymal stem cells (MenSCs), a type of adult stem cell, have gained attention as an appealing source for clinical applications due to their potential for structure regeneration, with ease of acquisition and regardless of ethical issues.

**Methods:**

The differential potential of MenSCs cocultured with AF cells was examined by the expression of collagen I, SCX, and CD146 using immunofluorescence. Western blot and ELISA were used to examine the expression of TGF-β and IGF-I in coculture system. An AF defect animal model was established in tail disc of Sprague-Dawley rats (males, 8 weeks old). An injectable gel containing MenSCs (about 1*10^6^/ml) was fabricated and transplanted into the AF defects immediately after the animal model establishment, to evaluate its repairment properties. Disc degeneration was assessed via magnetic resonance (MR) imaging and histological staining. Immunohistochemical analysis was performed to assess the expression of aggrecan, MMP13, TGF-β and IGF-I in discs with different treatments. Apoptosis in the discs was evaluated using TUNEL, caspase3, and caspase 8 immunofluorescence staining.

**Results:**

Coculturing MenSCs with AF cells demonstrated ability to express collagen I and biomarkers of AF cells. Moreover, the coculture system presented upregulation of the growth factors TGF-β and IGF-I. After 12 weeks, discs treated with MenSCs gel exhibited significantly lower Pffirrmann scores (2.29 ± 0.18), compared to discs treated with MenSCs (3.43 ± 0.37, *p* < 0.05) or gel (3.71 ± 0.29, *p* < 0.01) alone. There is significant higher MR index in disc treated with MenSCs gel than that treated with MenSCs (0.51 ± 0.05 vs. 0.24 ± 0.04, *p* < 0.01) or gel (0.51 ± 0.05 vs. 0.26 ± 0.06, *p* < 0.01) alone. Additionally, MenSCs gel demonstrated preservation of the structure of degenerated discs, as indicated by histological scoring (5.43 ± 0.43 vs. 9.71 ± 1.04 in MenSCs group and 10.86 ± 0.63 in gel group, both *p* < 0.01), increased aggrecan expression, and decreased MMP13 expression in vivo. Furthermore, the percentage of TUNEL and caspase 3-positive cells in the disc treated with MenSCs Gel was significantly lower than those treated with gel alone and MenSCs alone. The expression of TGF-β and IGF-I was higher in discs treated with MenSCs gel or MenSCs alone than in those treated with gel alone.

**Conclusion:**

MenSCs embedded in collagen I gel has the potential to preserve the disc structure and prevent disc degeneration after discectomy, which was probably attributed to the paracrine of growth factors of MenSCs.

**Supplementary Information:**

The online version contains supplementary material available at 10.1186/s13287-024-03680-w.

## Introduction

Disc herniation in the lumbar spine is a common cause of sciatica, which can now be effectively treated with endoscopic discectomy [1]. However, discectomy alone only addresses the removal of the herniated disc that is compressing the nerve root, neglecting the crucial aspect of structural repair and the inherent risk of further disc degeneration [2]. As a result, patients may continue to suffer from disabling back pain and face the possibility of re-herniation, which may require more invasive revision surgery.

To overcome these complications and enhance surgical efficacy while reducing medical costs, an innovative strategy applied during discectomy is needed. Regenerative medicine and tissue engineering have paved the way for a promising approach: the transplantation of stem cells combined with biological agents to repair the disc structure and restore its physiological function [3–5]. Animal studies have demonstrated that mesenchymal stem cells (MSCs) possess the capability to differentiate towards chondrogenic and nucleus pulposus (NP) cell types, promoting extracellular matrix (ECM) production, and decreasing the apoptosis of resident cells in disc [4, 6, 7]. Moreover, MSCs exhibit inherent immunomodulatory capacities, potentially mitigating the inflammatory environment within the intervertebral disc upon transplantation [8, 9]. Notably, numerous clinical trials have indicated substantial improvements in pain and function upon intradiscal injection of both adipose-derived MSCs (ADSCs) [10] and bone marrow-derived MSCs (BMSCs) [11, 12]. Particularly noteworthy are recent randomized controlled trials where a single intradiscal injection of allogeneic mesenchymal precursor cells resulted in a significant improvement of life quality [13].

While stem cells hold great promise for intervertebral disc repair, the choice of seed cells has become a bottleneck for clinical applications. BMSCs and ADSCs have been extensively studied in animal models [3, 4, 7]. However, limitations such as donor site trauma, ethical issues, and long doubling time hinder their application in human beings [14, 15]. Therefore, an ideal candidate for regenerative medicine should exhibit easy accessibility, stable performance, and ethical compliance.

Menstrual blood-derived mesenchymal stem cells (MenSCs), isolated from fragments of endometrial tissue in menstrual blood, are increasingly being applied in regenerative medicine [16–19]. MenSCs display typical stromal fibroblast morphology and exhibit rapid propagation, with higher telomerase activity than BMSCs [20]. Various studies have demonstrated the regenerative capacity and functional recovery potential of MenSCs in myocardial infarction [21], Duchenne muscular dystrophy [22], and acute liver failure [23]. Recent research conducted by our group has also indicated the beneficial effects of MenSCs transplantation in acute lung injury [24] and liver fibrosis [25]. The ease of acquisition, regardless of ethical issues, and short doubling time make MenSCs an appealing source for clinical applications. Emerging data support the application of MenSCs for cartilage and rotator cuff regeneration in animal models, demonstrating protective effects on ECM and tissue healing [26, 27]. However, the understanding of MenSCs’ capacity for disc repair and the associated mechanisms remains limited.

Previous studies have indicated that MenSCs can differentiate into NP-like cells when cocultured with NP cells [28]. The mending of annulus fibrosis (AF) is crucial for closing defects post-discectomy and diminishing the likelihood of recurrence. It is intriguing to explore whether MenSCs could differentiated into AF-like cell in vitro, and serve as a source of seed cells for repairing AF defects. On the other hand, the survival and adaptability of MenSCs within the intervertebral disc necessitate thorough examination [19, 29]. A wealth of evidence suggested that their reparative effects in vivo appear to stem more from paracrine actions than differentiation and tissue integration [19, 30, 31]. Consequently, it is meaningful to examine the paracrine effects of MenSCs in disc defects.

As is well-known, the collagen I is the main composition of AF, especially the outer layer. In addition, collagen I gel is a good carrier biomaterial for lots of organ and tissue regeneration, as demonstrated by recent researches [32–34]. Its fiber and three-dimensional porous structure are benefit to the growth of AF cells [32] and the permeability of growth factors [35, 36]. The animal model of AF defect was created by the knife-edge of needle, to imitate the irregular breach of AF after discectomy. The feature of gel can well fulfill with the irregular defect.

Therefore, our study aimed to investigate the effects of transplanting MenSCs embedded in collagen I gel on the repair of disc defects and the prevention of ongoing disc degeneration following discectomy.

## Materials and methods

### Preparation of MenSCs

MenSCs were obtained from the Innovative Precision Medicine Group (IPM, Hangzhou, China). Volunteers between the ages of 20 and 45 were educated about the donation process and provided signed informed consent, following procedures outlined in previous studies [16, 37]. These cells underwent rigorous testing for characteristics, cell cycle, verification of undifferentiated state, and multipotent differentiation ability in osteogenic, chondrogenic, and adipogenic lineages [16, 37]. The biomarkers of stem cells were examined by flowcytometry, revealing positive staining for CD29, CD73, and CD90 (**Fig. ****S1**** A-C**), and negative staining for CD11b, CD19, CD45, CD34, HLA-DR, and stage-specific embryonic antigen (SSEA)-4 (**Fig. ****S1**** D-I**).

### Co-culture of MenSCs with AF cells

**Fig. 1:**
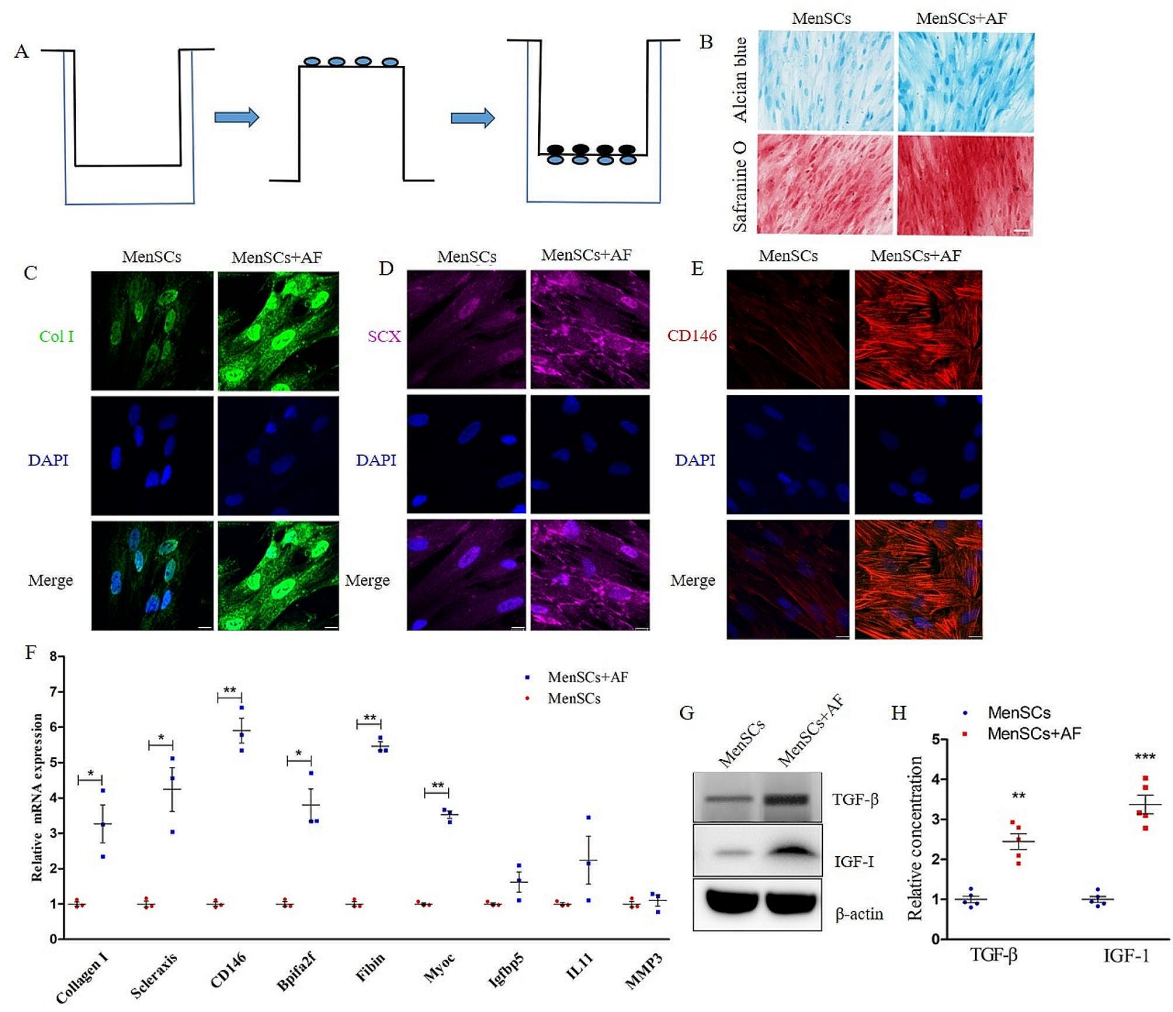
AF cells and MenSCs co-culture promote production of extra-cellular matrix and growth factors. (**A**): The coculture system of MenSCs and AF cells; (**B**) Alcian blue and safranine O staining; (**C**) collagen I immunofluorescence; (**D**): Scleraxis immunofluorescence;(E): CD146 immunofluorescence; (**E**) qPCR for collagen I, Scleraxis, CD146, Bpifa2f, Fibin, and Myoc, Igfbp5, MMP3, and IL-11. (**F**) Western blot for TGF-β and IGF-I; (**G**) ELISA for TGF-β and IGF-I. Values are presented as mean ± SEM. ** *P* < 0.01, *** *P* < 0.001. Col I, collagen I; SCX, scleraxis; MenSCs, menstrual blood-derived mesenchymal stem cells; AF, anulus pulposus. Scale bar = 20 μm for immunofluorescence, scale bar = 50 μm for safranine O staining

MenSCs were cultured according to the manufacturer’s instructions, with medium replacement occurring twice weekly. For our experiment, MenSCs at passage 2 were utilized. The co-culture of MenSCs and AF cells (Passage 2) was performed by seeding MenSCs in six-well plates and growing AF cells in culture inserts (Becton Dickinson Labware, NJ, USA) in a cell-to-cell contact manner, as demonstrated in Fig. 1A. The culture inserts contained a 0.4 μm pore-size filter, enabling communication between these two cell types. Firstly, 1 × 10^4^ AF cells were cultured on the reverse membrane of the insert for 8 h for cells adhesion. Secondly, the insert was turned upright and another 1 × 10^4^ MenSCs were placed on the front membrane of the insert. AF cells and MenSCs were maintained in Dulbecco’s Modified Eagle Medium (DMEM) /F-12 with 10% fetal bovine serum for 1 weeks at 37 °C in a humid atmosphere containing 5% CO_2_. The culture medium was replaced every 2 days. The experimental groups were as follows: (a) MenSCs mono-culture; (b) AF cells + MenSCs co-culture. MenSCs were harvested and processed for qPCR analysis or histochemical analysis, while the supernatant was collected for ELISA assay.

### Preparation of collagen I gel combined with MenSCs

Collagen I gel (Rat tail, Shengyou, Hangzhou, China) was utilized as an alginate scaffold for MenSCs. The collagen I was stored at a concentration of 4 mg/ml and was dissolved in NaOH and phosphate-buffered saline (PBS). For the preparation of 1 ml collagen I hydrogels (2 mg/ml), 500 µl of the original collagen I fluid was first diluted in 376 µl of ddH_2_O. Subsequently, 24 µl of NaOH (1 mol/L) was added to the mixture. Finally, 100 µl of 10X PBS was added to form the gel. For the preparation of 1 ml collagen I hydrogels combined with MenSCs, 500 µl of the original collagen I fluid was mixed with 24 µl of NaOH (1 mol/L) in a 15 mL polypropylene tube. Then, 46 µl of 10X PBS and 430 µl of the cell suspension were added to form a three-dimensional cell gel. The final cell density was about 1.0*10^6^ cells/mL. The hydrogels were formed in a humidified incubator at 37℃ and 5% CO_2_ for 1 h, and DMEM was added for cell culture.

### Cell proliferation and cytotoxicity assessment

To assess the biocompatible characteristics of MenSCs and collagen I, the Cell Counting Kit-8 (CCK8, Beyotime, C0037) and lactic dehydrogenase (LDH) assay kit (Beyotime, C0016) were utilized following the manufacturer’s instructions. Firstly, MenSCs were combined with collagen I gel, as described previously. Aliquots of 100 µL were then seeded onto polypropylene tubes. DMEM was carefully overlaid onto each gel, and the medium was refreshed every 2 days. MenSCs formed three-dimensional micro-masses within the polypropylene tubes were considered as control. To conduct the CCK8 analysis, 500 µL of DMEM was replaced with fresh medium, followed by the addition of 50 µL of CCK-8 solution for a 4-hour incubation period. Subsequently, 100 µL of the samples was collected and measured in a 96-well plate using a Model 680 microplate reader (Bio-Rad, Hercules, CA) at 450 nm. The release of LDH in the supernatant was measured using a microplate reader in a 96-well plate at 450 nm. Positive controls were established using cells treated with Triton X-100. LDH activity was calculated as the percentage of the experimental group relative to the positive control.

### qPCR

Total RNA was extracted from the cells using TRIzol reagent (Takara Bio, Inc.). RNA was reverse transcribed into cDNA using the RevertAid First Strand cDNA Synthesis kit (Takara Bio, Inc.). The cDNA was then amplified with a Real-time PCR System (7900; Applied Biosystems, USA) and a SYBR Green PCR Master Mix (Takara, Japan). The conditions for the real-time PCR amplification reaction were 95˚C for 10 s, 92˚C for 30 s, 60˚C for 38 s and 72˚C for 42 s, 40 cycles in total. The dissolution curve was analyzed to determine the specificity of the real-time PCR amplification The Ct (cycle threshold) values, the number of PCR cycles to reach a setting threshold, was obtained for each sample. Quantification of the relative expression levels of these target genes was achieved by the following formula. 2^-ΔΔCt^, where ΔΔCt = (Ct of the target gene - Ct of β-actin)_treatment_-(Ct of the target gene - Ct of β-actin)_control_. Data were given in arbitrary units relative to control, which were defined as value 1. The primers of the related genes are listed in the Supplement Table, and β-actin was used as internal control.

### ELISA analysis

The supernatant samples of coculture systems were collected and then centrifuged at 2000 g for 10 min to protein enrichment. The concentrations of TFG-β and IGF-I were determined using ELISA kits (Biorbyt, San Francisco, California, USA), following the instructions of manufacturer. All samples were performed in triplicate.

### Alcian blue staining

After 2 weeks of coculture, MenSCs from each group were transferred into 6 well plates by 0.25% trypsin digestion and cultured for 1 day. Then MenSCs were fixed in 4% paraformaldehyde. The fixed samples were washed with PBS and stained with 0.5% Alcian blue in 3% acetic acid for 30 min. The cells were visualized and photomicrographed with a light microscopy (Leica DM 2500).

### Immunofluorescence

MenSCs were cultured in 20 mm glass-bottomed dishes (Nest Biotechnology, Shanghai, China). The cells were fixed with 4% paraformaldehyde for 15 min, and washed two times with PBS containing 0.05% Tween-20, permeabilized with 0.3% Triton X-100 for 5 min, and then blocked with 1% BSA for 30 min. Then dishes were incubated with collagen I (1:200, Abcam, ab138492), Scleraxia (1:150, Abcam, ab138492) and CD146 (1:200, Abcam, ab228540) antibodies at 4 °C overnight. The dishes were then incubated with goat anti-rabbit IgG conjugated to fluorescent cy3 dye (1:100; Boster, Wuhan, China). The nuclear was stained with 4ʹ,6-Diamidino-2-phenylindole (DAPI, Beyotime, C1006, shanghai, China). The images were captured using an Olympus confocal microscope and processed with Image-Pro Plus 6.0 (NIH, Bethesda, USA).

### Animal study

Sprague-Dawley rats (males, average weight 200 g; 8 weeks old, Zhejiang Academy of Medical Sciences, Hangzhou, China) were used in our study. The rats were clean animal without any previous procedures and any immune problems, and they were kept under clean conditions with appropriate temperature, humidity, and free access to sterilized water and pellet food. The protocol was approved by the institutional review board at our institution, and all the procedures were conducted following the Guidance for the Care and Use of Laboratory Animals.

The rats were anesthetized using sodium pentobarbital (10 mg/kg) administered via intraperitoneal injection. Initially, the tail disc was marked on the body surface using a marker pen, and a tourniquet was tied at the base of the tail to minimize blood loss. A 0.5 cm longitudinal sagittal incision was made along with the tail (**Fig. ****S2****A**). Subcutaneous soft tissues were dissected and detached to expose the surface of AF (**Fig. ****S2****B**). The disc defect was created using a 22-gauge needle with an outer diameter of 0.7 mm (**Fig. ****S2****C**). The needle’s knife-edged angular surface was capable of cutting AF and creating a defect in the disc. Gel transplantation occurred following the establishment of AF defects, with injection along the break of AF to effectively fill the defect. Immediate closure of the subcutaneous layer and skin followed (**Fig. ****S2****D, E**). Tramadol was given after the surgical procedure for pain relief.

Fourteen rats were used in our experiments. The grouping was illustrated in **Fig. ****S2****F**, and different treatments were performed in the same rat at different disc levels. The disc defect treated with DMEM was designated as the vehicle control (Discectomy + Vehicle group), In the Discectomy + Gel group, disc defects were filled with 200 µl of 2% collagen gel. In the Discectomy + MenSCs group, disc defects were filled with MenSCs solution using a microsyringe (Hamilton Medical, Bonaduz, Switzerland). In the Discectomy + MenSCs + Gel group, disc defects were filled with MenSCs embedded in collagen gel using a microsyringe. The disc without any procedure served as the intact control. After conducting preliminary experiments, it was determined that 2 ml of collagen I gel combined with MenSCs was required for 7 discs, while 2 ml of collagen I gel without MenSCs was used for the other 7 discs as a control. The animal experiment was performed by one author (XJH) in one time, to minimize potential confounders. Each three rats were randomly housed in a cage, and kept in the same area of animal room. The rats were daily monitored, and any rats with incision infection or weight loss were excluded for any assessments. At 4 and 12 weeks postoperatively, each 7 rats were euthanized by an overdose sodium pentobarbital anaesthesia (40 mg/kg) via intraperitoneal injection.

### Radiological studies

The rat’s tails were then imaged using a 3.0-Tesla scanner (MR, Philips, the Netherlands). T2-weighted (T2W) images were acquired with a TE (time of echo)/TR (time of repetition) of 99/2180 ms, a matrix size of 256*256, and a field of view of 90*90. The slice thickness was 2 mm, and there was an intersection gap of 0.3 mm. Signal intensity measurements were performed on T2W midsagittal images using DICOM Viewer (Version 3.0; Philips, Netherlands). The MR index was calculated as ratios of the target disc signal intensity relative to that of the intact control in the same rat [4].

### Histological studies

The tails of all rats were decalcified in 10% ethylenediaminetetraacetic acid for a period of 2 weeks. Using a pathological anatomical knife, the tails were cut into two sagittal halves along the middle line of the scar, and serial 5 μm sagittal sections were obtained for histological examinations. Hematoxylin and eosin (HE) staining and safranin O-fast green staining were performed to assess the severity of disc degeneration.

The histological examinations of the discs were conducted using the final consensus degeneration grading system, which consists of five categories and ranges from 0 to 16 points [38]. This grading system incorporates a comprehensive list of morphological categories and features, enabling the evaluation of different degrees of disc degeneration. The assessment includes the evaluation of NP morphology, NP cellularity, AF morphology, NP-AF border, and endplate condition. Noteworthy features such as alterations in notochordal cells, NP morphology, and AF organization were given double weighting, with two features contributing to the overall score. Thus, the grading system encompasses a total of eight degenerative features, each scored on a 3-point scale (0–2), resulting in an overall score ranging from 0 to 16. Severely degenerated intervertebral discs exhibited characteristic features such as reduced NP volume, decreased NP cellularity, ruptured AF, and disorganized endplate. These findings were indicative of advanced disc degeneration.

### Immunohistochemistry

Biochemical changes, including catabolism and anabolism, were examined through immunohistochemistry. The tissue sections were first dewaxed and brought to water, followed by incubation in 3% H_2_O_2_ for 15 min to block endogenous peroxidase activity. Subsequently, the slices were washed with PBS and then subjected to antigen retrieval by immersion in boiling citrate buffer for 1 min.

After blocking with 5% bovine serum albumin for 20 min to prevent nonspecific binding, the slices were incubated with primary antibodies against aggrecan (1:1000, Omnimabs, OM197094), MMP-13 (1:1000, Omnimabs, OM284898), TGF-β ((1:500, Abcam, ab215715), and IGF-I (1:50, Abcam, ab106836). This incubation step took place overnight at 4 °C in a wet box to facilitate antibody binding. On the following day, the primary antibody was removed, and the sections were washed repeatedly with PBS.

Subsequently, the slices were incubated with a horseradish peroxidase-labeled secondary antibody (1:1000, Boster, BA1054, Wuhan, China) for 30 min at room temperature. This secondary antibody binds specifically to the primary antibody, allowing for the visualization of the target protein. The expression of the target protein was visualized by the addition of diaminobenzidine solution, resulting in a brown color reaction. To enhance contrast, the sections were re-stained with hematoxylin.

Observation of the immunohistochemical staining was performed using an Olympus microscope, and a computer-aided image acquisition system was employed for quantification analysis. Three fields were randomly selected (at 400-fold magnification) from each tissue slice to count the number of positive cells expressing the target protein. All experiments were performed on seven slices in each group.

### TUNEL assay

Cell apoptosis in the disc was examined using the terminal deoxynucleotidyl transferase-mediated dUTP nick-end labeling (TUNEL) technique, employing the in situ cell death detection kit from Roche Diagnostics (Mannheim, Germany). The tissue sections were first permeabilized by treating them with 20 mg/mL proteinase K (Meck & Millpore, USA) for 1 h, followed by washing with PBS. Next, the sections were incubated with the TUNEL reaction mixture at 37 °C in a dark and humid box for 1 h. The TUNEL reaction detects DNA fragmentation, a characteristic of apoptotic cells. After incubation, the sections were washed with PBS and then stained with 4’,6-diamidino-2-phenylindole (DAPI, Beyotime, C1006) as a counterstain. Finally, the sections were sealed with an anti-fluorescence quenching solution.

To observe the TUNEL-positive cells, a confocal microscope (Olympus, Japan) was used. The positive cells were counted at a magnification of 400 in three fields of each slide. The ratio of cell apoptosis was determined by calculating the percentage of positive cells relative to the total cell count in the observed fields. All experiments were performed on seven slices in each group.

### Statistical analysis

Statistical analyses were conducted using STATA software (version 13.0; Stata Corp, USA). The data are presented as means ± standard error of the mean (SEM). To analyze the differences in parameters such as Pfirrmann scores, MR index, histological scores, and percentage of positive cells among the various groups, one-way analysis of variance (ANOVA) was performed. Data analysis was performed by an author who was blinded to experimental protocol. Post hoc analysis was carried out using Turkey test. The level of statistical significance was set at *p* < 0.05.

## Results

### AF cells and MenSCs co-culture promoted MenSCs expressing bio-markers of AF cells

MenSCs were co-cultured with AF cells to assess their differential potential (Fig. 1A). Collagen I and aggrecan, key components of the ECM secreted by AF cells, were examined. Safranine O staining demonstrated increased aggrecan staining in MenSCs co-cultured with AF cells (Fig. 1B). Immunofluorescence analysis revealed significantly higher expression of collagen I in MenSCs co-cultured with AF cells compared to MenSCs alone (Fig. 1C).

Moreover, the expression of Scleraxia (SCX) and CD146, biomarkers of AF cells, was higher in MenSCs co-cultured with AF cells (Fig. 1C-E). In addition, the gene expression levels of collagen I, SCX, and CD146, along with the novel biomarkers of AF as reported by J. Wang [39] (Bpifa2f, Fibin, and Myoc), upregulated significantly when cocultured with AF cells, while no significant changes were observed in Igfbp5, MMP3, and IL-11(Fig. 1F). The co-culture system also showed upregulation of TGF-β and IGF-I, as indicated by Western blot (Fig. 1G) and ELISA (Fig. 1H) assays, suggesting the paracrine effects of MenSCs.

### Biocompatibility of MenSCs and collagen gel

The biocompatibility of MenSCs and collagen gel was evaluated using CCK8 and LDH release assays. The cell number of MenSCs in medium containing collagen gel was found to be similar to that of MenSCs cultured in the standard medium, indicating that the proliferation of MenSCs was not affected by the presence of the collagen gel (**Fig. ****S3****A**). Furthermore, there was no significant difference in LDH release between the two groups (**Fig. ****S3****B**), indicating that the collagen gel or vehicle did not exhibit any toxicity towards the MenSCs. Scanning electron microscopy revealed that the collagen I scaffold exhibited lamellar structures, forming a three-dimensional scaffold with numerous pores, most of which had a diameter exceeding 50 μm (**Fig. ****S3**** C-F**). These findings suggested that collagen I gel have good biocompatibility and well-suited for MenSCs penetration and adhesion within the disc.

### MenSCs embedded in collagen gel preserved the water content of degenerated disc following discectomy (*N* = 7)

**Fig. 2:**
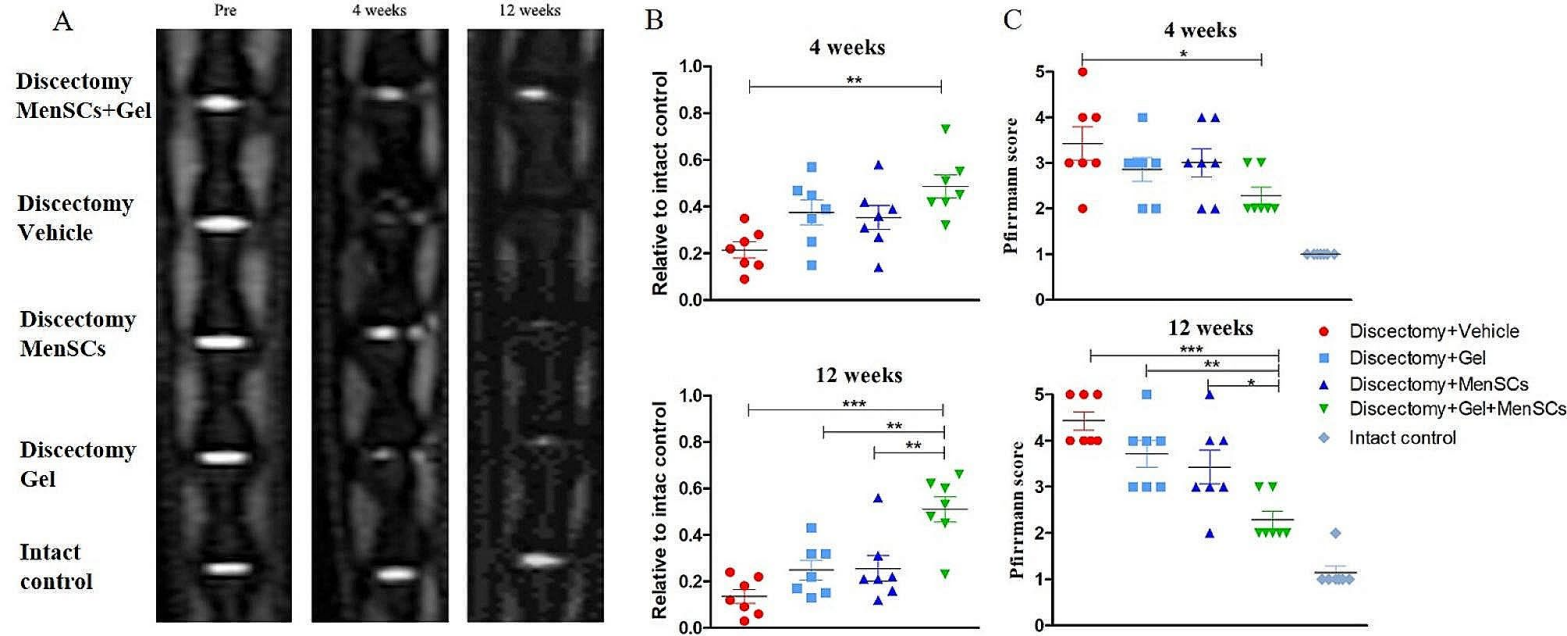
MenSCs embedded in collagen gel preserved the water content of degenerated disc following discectomy. (**A**): Midsagittal MR images (T2- weighted) of degenerated disc at 4 and 12 weeks after various treatment. Images are representative of seven replicates. (**B**) MR imaging index for each group. (**C**): Pfirrmann grading of disc degeneration after various treatment. Values are presented as mean ± SEM. * *P* < 0.05, ** *P* < 0.01, *** *P* < 0.001. MenSCs, menstrual blood-derived mesenchymal stem cells. MR, magnetic resonance

The degree of disc degeneration in different treatment groups was quantitatively analyzed using MRI index and qualitatively assessed using the Pfirrmann score based on midsagittal T2-weighted MR images (Fig. 2A). The MR images revealed that the signal intensity of discs repaired with MenSCs + Gel was significantly higher compared to discs treated with the vehicle, gel alone, or MenSCs alone (Fig. 2A). The MRI index, which indicated the water content of the disc, was significantly higher in the MenSCs + Gel group compared to the vehicle group at both 4 weeks (0.49 ± 0.05 vs. 0.21 ± 0.03, *p* < 0.01, Fig. 2B) and 12 weeks (0.51 ± 0.05 vs. 0.13 ± 0.03, *p* < 0.001, Fig. 2B) postoperatively. Furthermore, the MRI index in the MenSCs + Gel group was also higher than that in the Gel group (0.51 ± 0.05 vs. 0.24 ± 0.04, *p* < 0.01) and the MenSCs group (0.51 ± 0.05 vs. 0.26 ± 0.06, *p* < 0.01) at 12 weeks postoperatively (Fig. 2B).

At 4 weeks postoperatively, a significant difference of Pfirrmann score was observed only between the vehicle group and the MenSCs + Gel group (3.43 ± 0.37 vs. 2.29 ± 0.18, *p* < 0.01). By 12 weeks postoperatively, the average Pfirrmann score of the MenSCs + Gel group (2.29 ± 0.18) was significantly lower than that of the Gel group (3.71 ± 0.29, *p* < 0.01, Fig. 2C) and the MenSCs group (3.43 ± 0.37, *p* < 0.05, Fig. 2C). Additionally, the Pfirrmann score of the intact group was significantly lower than that of the other four groups (*p* < 0.001).

### MenSCs embedded in collagen gel preserved the structures and ECM contents of degenerated disc following discectomy (*N* = 7)

**Fig. 3:**
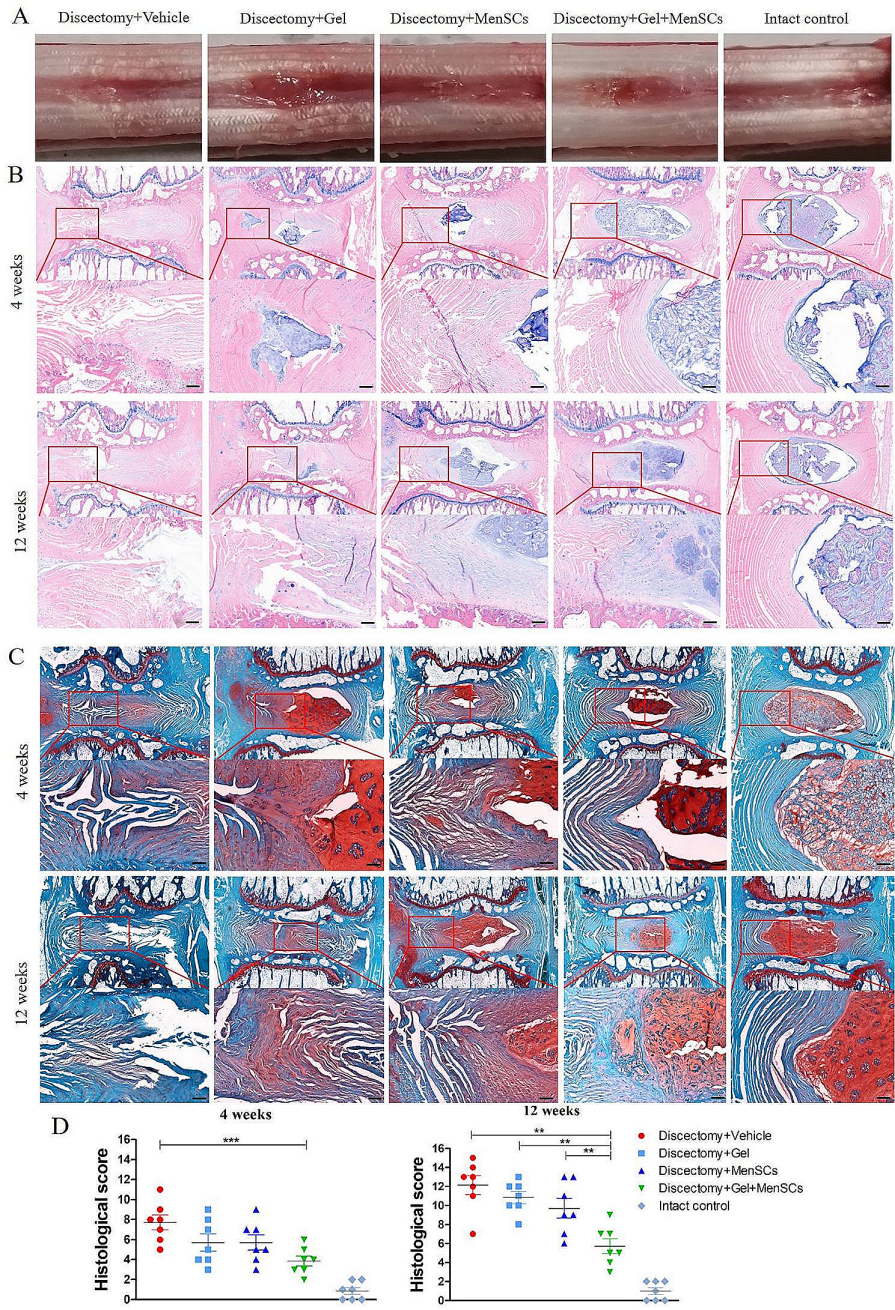
MenSCs embedded in collagen gel preserved the structures of degenerated disc following discectomy. (**A**): Gross appearance of the disc in different groups. (**B**): Hematoxylin and eosin staining of disc in different groups. (**C**): Safranin O and fast green staining of disc in different groups. (**D**): Disc degeneration scores. Images are representative of seven replicates. Scale bar = 100 μm; Values are presented as mean ± SEM. ** *P* < 0.01, *** *P* < 0.001

During dissection, a scar was observed on the surface of the annulus without any gel extrusion (**Fig. 34A**). There were no significant differences in the appearance of scars among the four groups. Histological evaluation of the discs showed that at 4 weeks postoperatively, the annulus fibrosus (AF) was disrupted and collapsed, and the nucleus pulposus exhibited fibrotic changes. By 12 weeks postoperatively, both the AF and NP degenerated severely in the discectomy control group (Fig. 3B, C). In contrast, the intact control group exhibited a typical oval-shaped NP tissue without structural collapse of the AF (Fig. 3B, C). Importantly, the disc structure was well-preserved in the MenSCs + Gel group throughout the follow-up period, with minimal fibrotic changes in the NP tissue and limited tearing of the annulus (Fig. 3B). Discs treated with either MenSCs or Gel alone demonstrated varying degrees of degeneration.

Accordingly, the histological score of disc degeneration in the MenSCs + Gel group (5.43 ± 0.43) was significantly lower than that in the vehicle group (12.14 ± 0.99), Gel group (10.86 ± 0.63), and MenSCs group (9.71 ± 1.04) at 12 weeks postoperatively (all *p* < 0.01, Fig. 3D). Furthermore, at 4 weeks postoperatively, there was a significant difference only between the Vehicle group and the MenSCs + Gel group (7.71 ± 0.75 vs. 3.86 ± 0.5, *p* < 0.05, Fig. 3D).

**Fig. 4:**
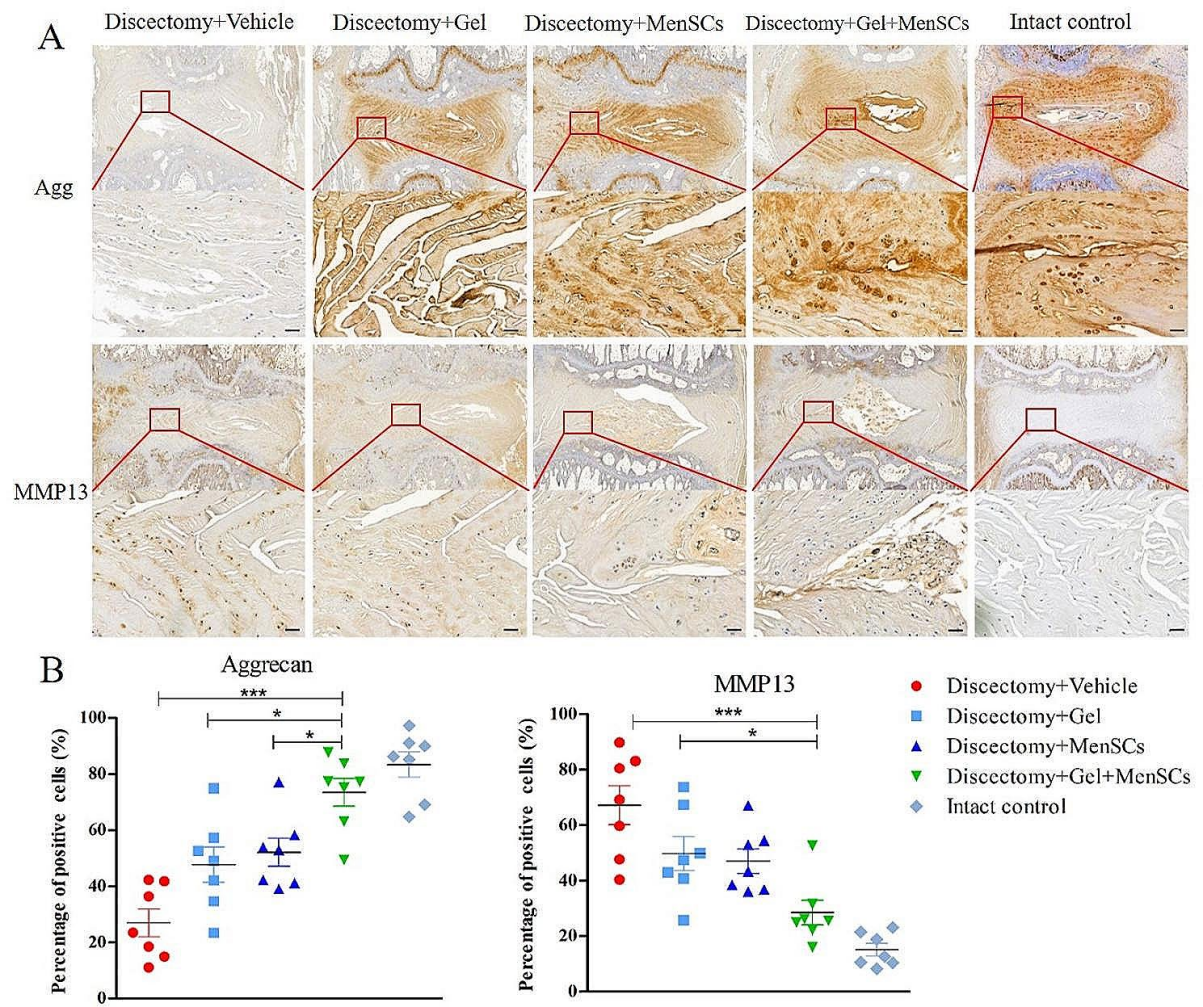
MenSCs embedded in collagen gel preserved extra-cellular matrix contents of degenerated disc following discectomy. (**A**): Immunohistochemical examination of aggrecan; (**B**): Immunohistochemical examination of MMP13; (**C**): Percentages of aggrecan and MMP13 positive cells relative to total cells in degenerated disc after discectomy. Images are representative of seven replicates. Scale bar = 50 μm; Values are presented as mean ± SEM. * *P* < 0.05, ** *P* < 0.01, *** *P* < 0.001. MenSCs, menstrual blood-derived mesenchymal stem cells, MMP3, matrix metalloproteinase 3

Immunohistochemical analysis was performed at 12 weeks to examine ECM production, with aggrecan (Fig. 4A) representing anabolism and MMP13 (Fig. 4B) representing catabolism. The percentage of aggrecan-positive cells in the MenSCs + Gel group was significantly higher compared to the vehicle, Gel, and MenSCs groups (all *p* < 0.001, Fig. 4C). In contrast, the percentage of MMP13-positive cells in the MenSCs + Gel group was significantly lower than that in the vehicle group (*p* < 0.0001) and the Gel group (*p* < 0.05), but not significantly different from the MenSCs group (Fig. 4C).

### MenSCs embedded in collagen gel up-regulated TGF-β and IGF-I expression in degenerated disc following discectomy (*N* = 7)

**Fig. 5:**
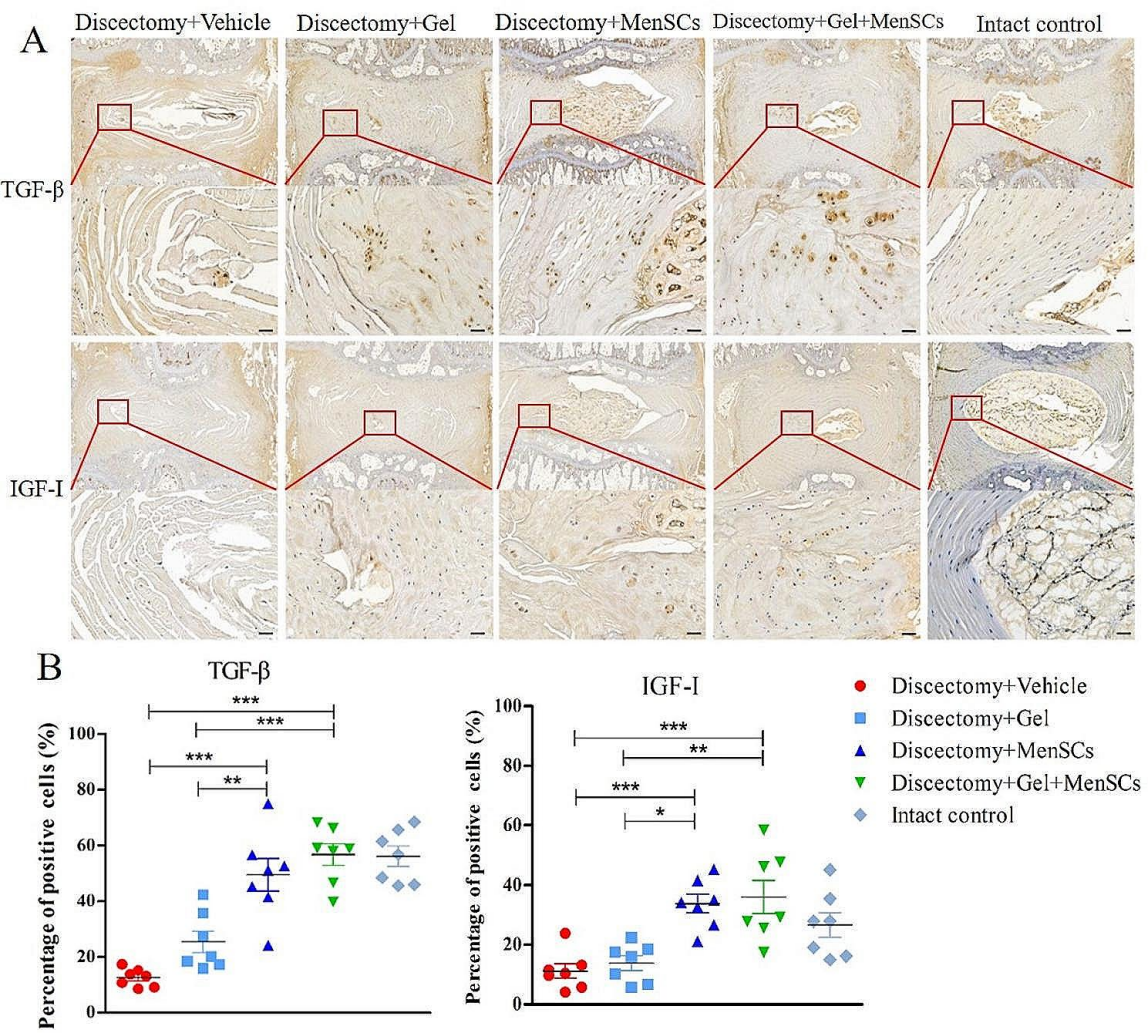
MenSCs embedded in collagen gel up-regulated TGF-β and IGF-I expression in degenerated disc following discectomy. (**A**): Immunohistochemical examination of TGF-β and IGF-I. (**B**): Percentages of TGF-β and IGF-I positive cells relative to total cells in degenerated disc after discectomy. Images are representative of seven replicates. Scale bar = 50 μm; Values are presented as mean ± SEM. * *P* < 0.05, ** *P* < 0.01, *** *P* < 0.001. MenSCs, menstrual blood-derived mesenchymal stem cells, TGF-β, transforming growth factor-β. IGF-I, insulin-like growth factor-I

The expression levels of growth factors, specifically TGF-β and IGF-I, were examined to assess the paracrine effects of MenSCs. Immunohistochemical assays demonstrated that the expression of TGF-β and IGF-I was predominantly localized at the site of repair in the designated area (highlighted by the red box in Fig. 5A). Moreover, the percentages of TGF-β and IGF-I positive cells in the MenSCs + Gel group and MenSCs group were significantly higher compared to the Vehicle group and Gel group. There was a significant difference of TGF-β expression between the MenSCs + Gel group and the MenSCs group, while no significant difference was observed for IGF-I expression (Fig. 5B).

### MenSCs embedded in collagen gel reduced the cell apoptosis in degenerated disc following discectomy (*N* = 7)

**Fig. 6:**
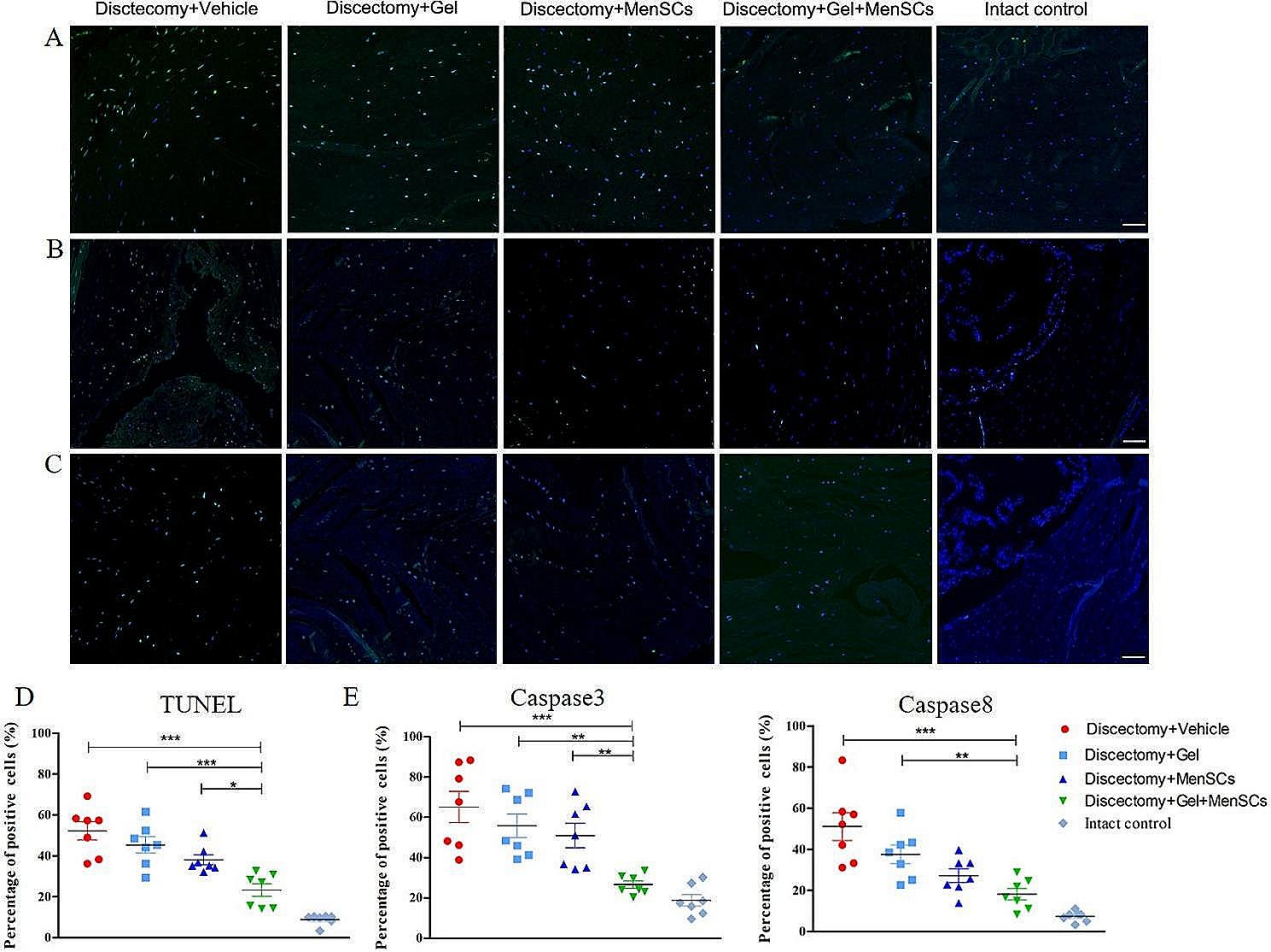
MenSCs embedded in collagen gel reduced the cell apoptosis in degenerated disc following discectomy. (**A**): TUNEL staining of apoptotic cells in the discs after various treatments. (**B**): Caspase 3 staining in disc after various treatments. (**C**): Caspase 8 staining in the discs after various treatments. Percentages of TUNEL (**D**), caspase 3 (**E**), and caspase 8 (**F**) positive cells relative to total cells. Images are representative of seven replicates. Scale bar = 200 μm; Values are presented as mean ± SEM. ** *P* < 0.01, *** *P* < 0.001. MenSCs, menstrual blood-derived mesenchymal stem cells. TUNEL, terminal deoxynucleotidyl transferase-mediated dUTP nick-end labeling

To assess apoptosis in the disc, the presence of TUNEL (Fig. 6A), caspase 3 (Fig. 6B), and caspase 8 (Fig. 6C) positive cells was evaluated (Fig. 6). In the sham control disc, approximately 18.8% of cells were TUNEL-positive. Interestingly, the percentage of TUNEL-positive cells in the disc treated with MenSCs + Gel (26.59%) was significantly lower compared to those treated with the vehicle (65.09%,), gel alone (55.77%), and MenSCs alone (50.95%) (Fig. 6D). It is worth noting that the percentage of TUNEL-positive cells in the MenSCs group was relatively lower than that in the Gel group (Fig. 6D). Furthermore, the percentage of caspase 3-positive in the disc treated with MenSCs + Gel (23.27%) was significantly lower compared to those treated with the vehicle (52.19%), gel alone (45.34%) and MenSCs alone (38.07%) (Fig. 6E). The percentage of caspase 8-positive in the disc treated with MenSCs + Gel (18.09%) was significantly lower compared to those treated with the vehicle (51.03%), gel alone (37.51%), but not MenSCs alone (27.19%) (Fig. 6F).

## Discussion

The surgical removal of a herniated disc can disrupt its stability and accelerate disc degeneration in the affected segment. Repairing the disc defects and preventing further degeneration pose significant challenges in the field. This study proposes a promising stem cell strategy for disc repair using MenSCs embedded in a collagen I gel, which has shown significant inhibition of disc degeneration in a discectomy model.

MenSCs, a promising stem cell resource derived from the cyclically regenerating human endometrium, have demonstrated similar characteristics to BMSCs [20]. They express typical MSC-associated markers and possess the ability to differentiate into various cell lineages. MenSCs have a higher proliferative capacity, with the ability to undergo 30–47 passages before senescence, compared to BMSCs at 20 passages [20]. Additionally, MenSCs exhibit distinctive immunomodulation properties and paracrine effects, enabling them to promote the endogenous cellular repair of the remaining cell population [17, 18].

Numerous studies have reported the regenerative capacity and functional recovery potential of MenSCs in various animal models and even clinical trials. MenSCs have been shown to promote liver structure regeneration and improve liver function in acute liver failure [23] and liver fibrosis [25] models. In a mouse model of type 1 diabetes mellitus [37], MenSCs enhanced the expression of neurogenin3 without differentiating into insulin-producing cells. MenSCs transplantation has also demonstrated positive effects in acute lung injury, pulmonary fibrosis, ischemic myocardial injury, and spinal cord injury models, attributed to the reduction of cell apoptosis, increased cell proliferation, upregulation of specific factors, and modulation of the inflammatory response [40]. Clinical studies involving MenSCs transplantation have been conducted in women with severe Asherman’s syndrome [41] and premature ovarian failure [42], leading to improved pregnancy rates and fertility, respectively.

In this study, MenSCs were found to secrete TGF-β and IGF-I while expressing ECM of the AF, similar as observed in an in vitro study [28]. However, the underlying mechanisms responsible for preserving disc structure and function remain unclear.

Despite the documented in vitro multi-differentiation capacity of MenSCs, it is worth noting that the reparative potential of MenSCs is primarily attributed to their paracrine effects rather than stem cell differentiation and integration into the tissue [43, 44]. Paracrine action, which involves the release of bioactive molecules such as growth factors, microRNAs, and lipids, plays a crucial role in tissue repair, micro-environment modulation, immunomodulation, and anti-apoptotic effects [45]. MenSCs secrete extracellular vesicles that act as carriers of these bioactive molecules, facilitating cellular repair and functional recovery [45]. In this study, the focus was on the secreted cytokines, including TGF-β and IGF-I, and the examination of cell apoptosis to demonstrate the anti-apoptotic effects of MenSCs. However, further analysis is needed to elucidate the specific actions of these growth factors.

To fully harness the potential of MenSCs for disc repair, an appropriate vehicle is required for cell delivery and to provide an optimal environment for tissue regeneration. Various hydrogels, such as polyethylene glycol, collagen I/II, and hyaluronic acid, have been utilized in MSC-based tissue engineering. Collagen gel, in particular, is an attractive option for disc regeneration due to its resemblance to the main ECM of the AF [32]. Its highly hydrated and three-dimensional porous structure, as shown by the SEM finding of our study **(**Fig. 3C-F), allows for the growth of MenSCs and appropriate permeability to growth factors [35, 36], such as TGF-β and IGF-I expressed by MenSCs.

Most tissue engineering studies of the disc focus on inhibiting degeneration caused by puncture injuries [46, 47]. However, it is important to consider that disc degeneration is not always synonymous with back pain, and certain therapeutic approaches may be ineffective in relieving symptoms or even lead to new traumatic degeneration. This study specifically investigated disc degeneration after discectomy and found that the implantation of MenSCs embedded in collagen gel could inhibit the degenerative process. Indeed, MenSCs were initially utilized in a murine model of Duchenne muscular dystrophy through cell fusion [22]. However, until 2023, their application in the musculoskeletal system remains limited. Recently, there is growing evidence showcasing their effectiveness in the repair of cartilage and tendons. Uzieliene et al. reported that MenSCs can stimulate a chondrogenic response in BMSCs by secreting activin A and TGF-β, potentially exerting protective effects on cartilage tissue by reducing the release of glycosaminoglycan [27]. Additionally, Song et al. suggested that MenSCs encapsulated in autologous platelet-rich gel led to the regeneration of more mature fibrocartilage at the healing site, facilitating rotator cuff healing in a rabbit model of chronic tears [26]. Notably, this is the first study reporting the application of MenSCs in disc degeneration repair. Findings revealed that MenSCs embedded in a collagen I gel have the potential to prevent disc degeneration after discectomy, possibly due to the paracrine effects of growth factors.

The use of commercially available MenSCs is a strength of this study, as it represents a readily available cell source for clinical applications. Furthermore, the study provides radiological and histological evidence supporting the beneficial effects of MenSCs embedded in collagen gel on ongoing disc degeneration after discectomy. However, there are certain limitations to consider. The protective mechanisms of MenSCs were not fully elucidated in this study, despite the observed overexpression of growth factors. We regrettably did not examine the biomarkers of MenSCs in the disc to confirm whether the cells retained their original state or differentiated into AF cells. The paracrine cytokine examined in our study was limited to TGF-β and IGF-I, a Human growth factors array can be used to outline the whole picture of cytokines secreted by the MenSCs. While the quantitative calculation of positive immunohistochemical staining is commonly employed in scientific reports, it is acknowledged that this method possesses limitations when compared to the comprehensive gene and protein quantitative analysis of the tissue. Additionally, the animal model used in this study involved discectomy in the rat tail disc, which does not perfectly replicate the surgical procedure and dynamic environment of human disc degeneration. Further studies using alternative animal models may provide additional insights into the efficacy of MenSCs combined with collagen gel for disc repair after discectomy.

## Conclusion

In conclusion, the findings from this study provide compelling evidence that the combination of MenSCs and collagen I gel can effectively prevent disc degeneration and inhibit cell apoptosis following discectomy. This beneficial effect is probably mediated by the secretion of TGF-β and IGF-I by MenSCs. MenSCs offer several advantages, including easy acquisition, low immunogenicity, and potential paracrine effects, making them a promising strategy for disc repair after discectomy. However, further research is necessary to uneven the molecular mechanisms underlying the paracrine vesicles and elucidate the precise actions of MenSCs in disc repairment.

### Electronic supplementary material

Below is the link to the electronic supplementary material.

Supplementary Material 1

Supplementary Material 2

Supplementary Material 3

Supplementary Material 4

Supplementary Material 5

Supplementary Material 6

Supplementary Material 7

## Data Availability

All data generated or analyzed during this study are included in this published article. The datasets used and/or analyzed during the current study are available from the corresponding author on reasonable request.

## Abbreviations

MenSCs: Menstrual blood-derived mesenchymal stem cells
BMSCs: Bone marrow-derived mesenchymal stem cells
PBS: Phosphate-buffered saline
DMEM: Dulbecco’s Modified Eagle Medium
CCK8: Cell Counting Kit-8
LDH: Lactic dehydrogenase
HE: Hematoxylin and eosin
AF: Annulus fibrosus
NP: Nucleus pulposus
MMP: Matrix metalloproteinase
ECM: Extracellular matrix
TUNEL: Transferase-mediated dUTP nick-end labeling
DAPI: 4’,6-diamidino-2-phenylindole
SEM: Standard error of the mean
ANOVA: Analysis of variance
MR: Magnetic resonance
TGF-β: Transforming growth factor beta
IGF-I: Insulin-like growth factor-I
