# Supplementary figures and images for: Menstrual blood-derived mesenchymal stem cells combined with collagen I gel as a regenerative therapeutic strategy for degenerated disc after discectomy in rats
Source: Stem Cell Res Ther. 2024 Mar 13;15:75. doi: 10.1186/s13287-024-03680-w (PMC10935903; doi:10.1186/s13287-024-03680-w)

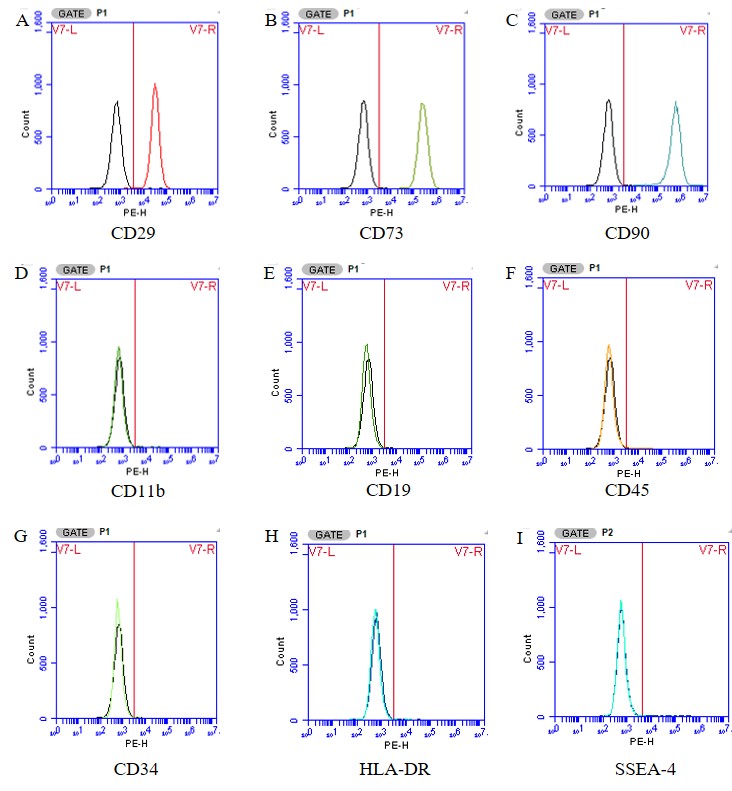

Supplement: Supplementary file 1 — Supplementary Material 1 [file 13287_2024_3680_MOESM1_ESM.jpg]

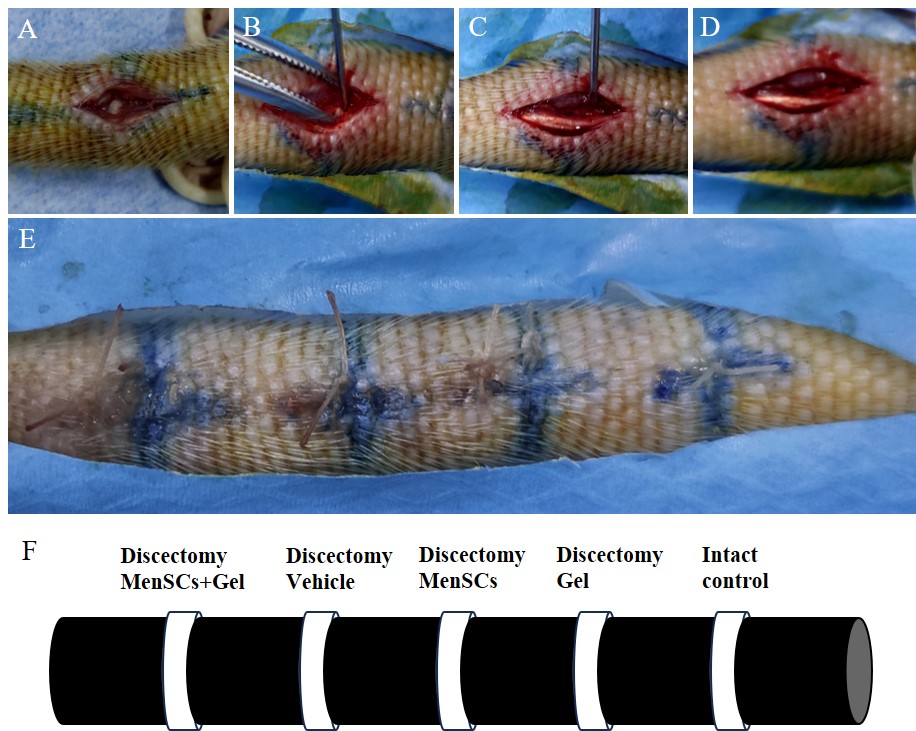

Supplement: Supplementary file 2 — Supplementary Material 2 [file 13287_2024_3680_MOESM2_ESM.jpg]

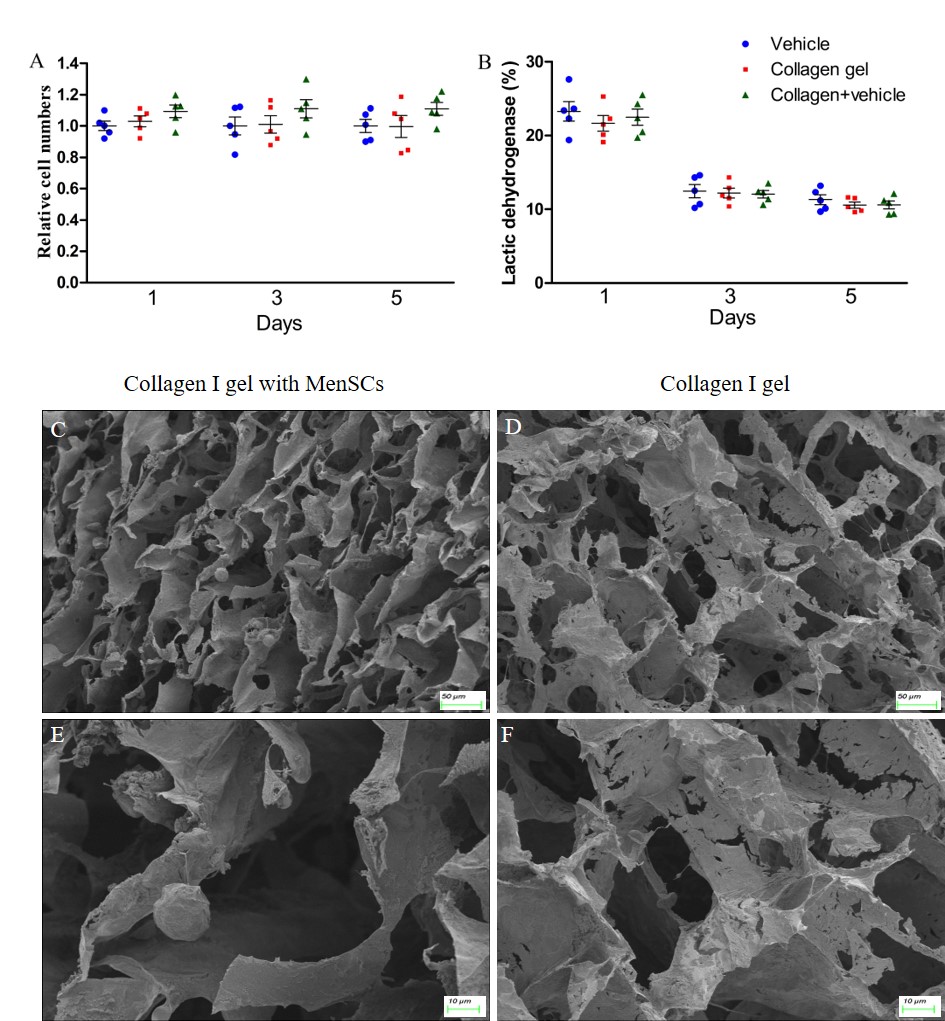

Supplement: Supplementary file 3 — Supplementary Material 3 [file 13287_2024_3680_MOESM3_ESM.jpg]

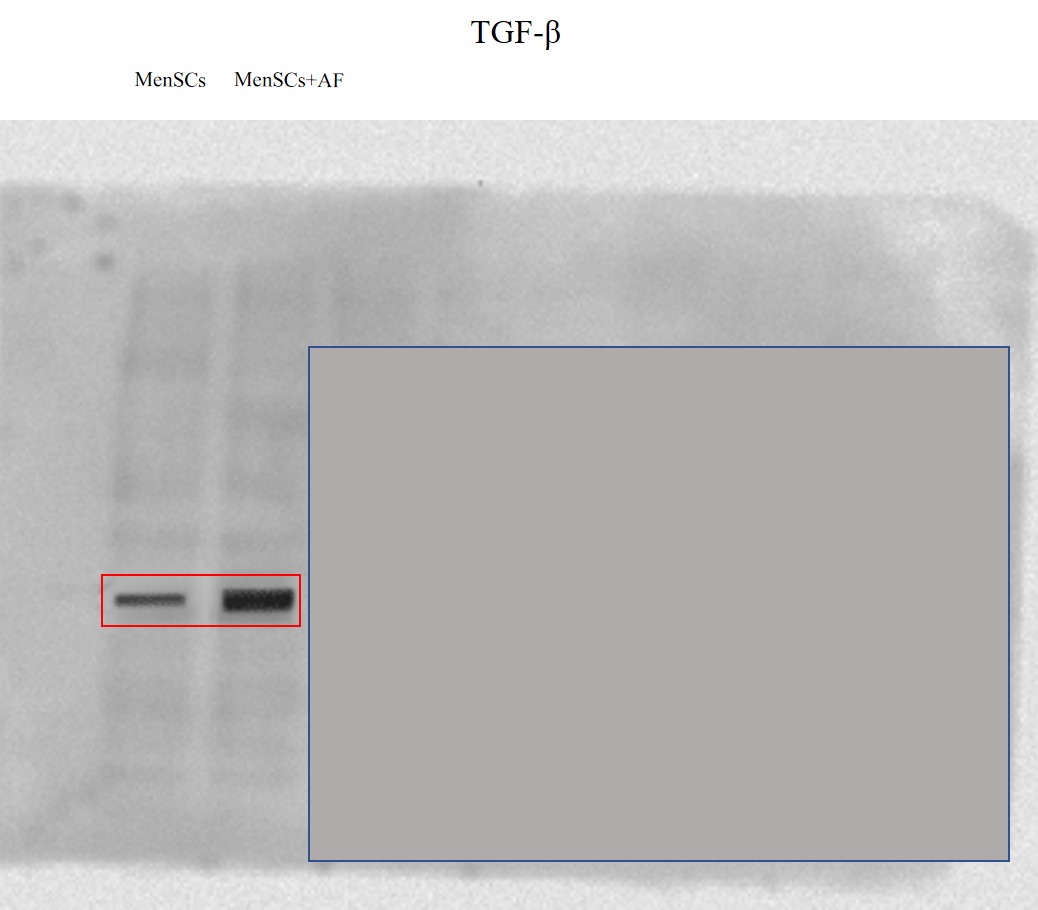

Supplement: Supplementary file 4 — Supplementary Material 4 [file 13287_2024_3680_MOESM4_ESM.jpg]

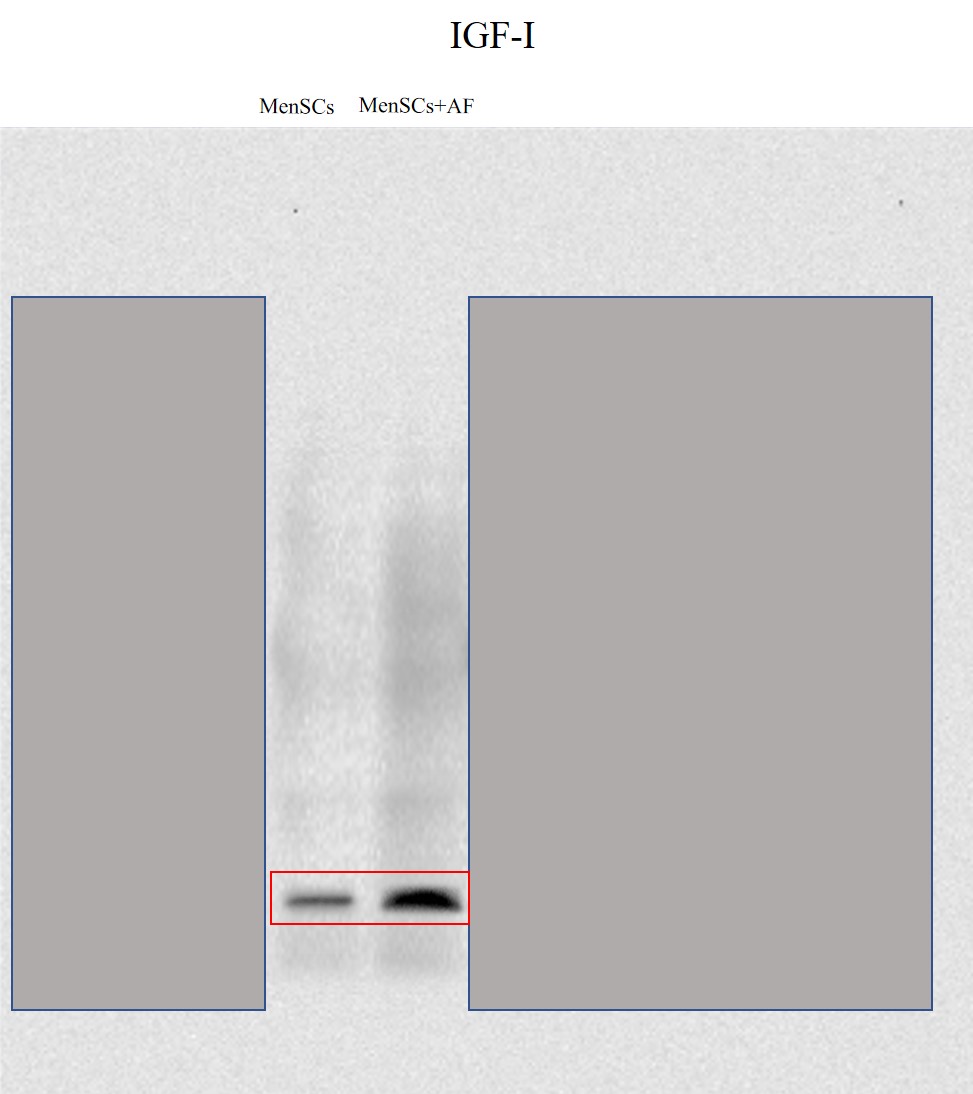

Supplement: Supplementary file 5 — Supplementary Material 5 [file 13287_2024_3680_MOESM5_ESM.jpg]

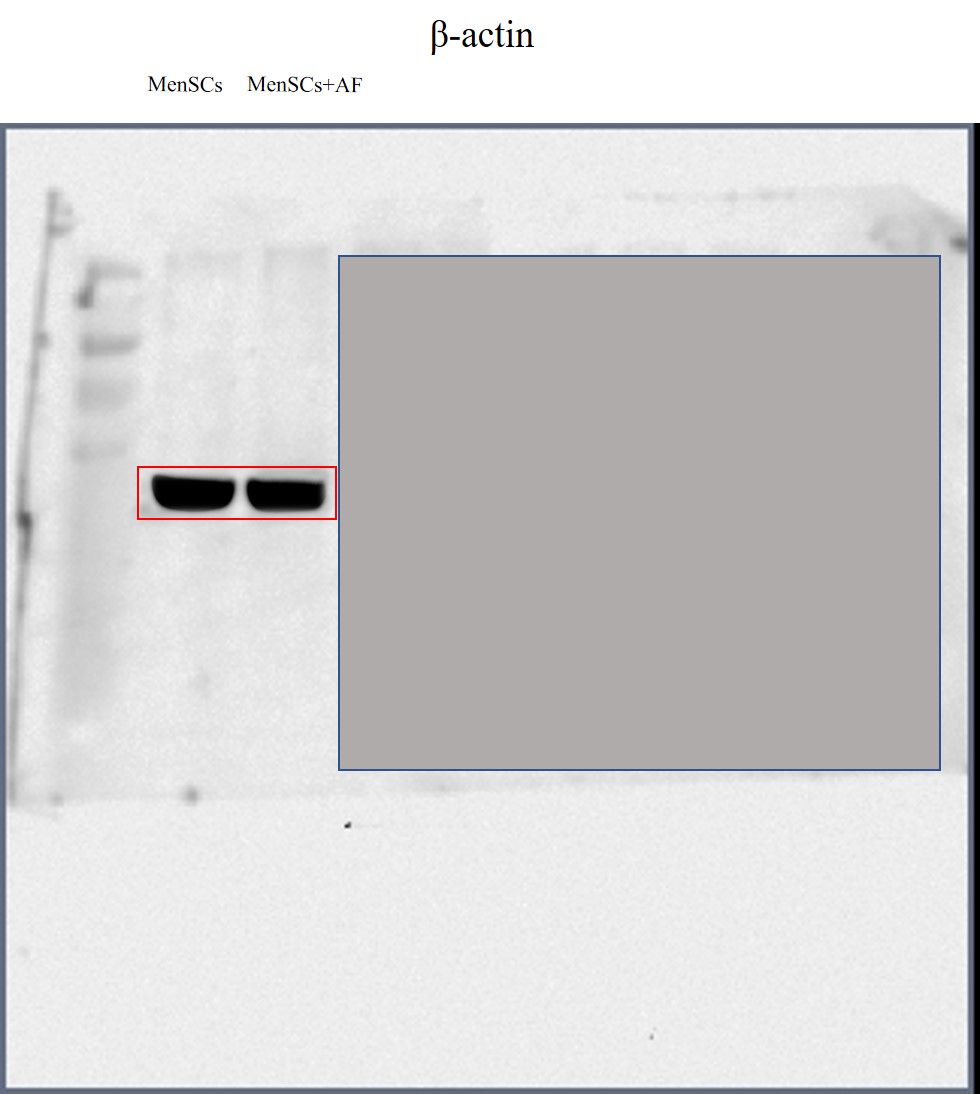

Supplement: Supplementary file 6 — Supplementary Material 6 [file 13287_2024_3680_MOESM6_ESM.jpg]
