# Supplementary material for: Menstrual blood-derived mesenchymal stem cells combined with collagen I gel as a regenerative therapeutic strategy for degenerated disc after discectomy in rats
Source: Stem Cell Res Ther. 2024 Mar 13;15:75. doi: 10.1186/s13287-024-03680-w (PMC10935903; doi:10.1186/s13287-024-03680-w)
Supplement: Supplementary file 7 — Supplementary Material 7 [file 13287_2024_3680_MOESM7_ESM.docx]

**Additional file 1: The biomarkers of MenSCs**

(A-C) Positive expression of CD29, CD73, and CD90 for MenSCs, revealed by flowcytometry; (D-I): Negative expression of CD11b, CD19, CD45, CD34, HLA-DR, and stage-specific embryonic antigen (SSEA)-4, revealed by flowcytometry.

**Additional file 2: The surgical procedures and design of the animal experiments**

(A): The incision of rat tails. (B): The exposure of surface of annulus fibrosis. (C): The defect created by a needle (arrowhead) (D): The disc defects treated with gel or MenSCs. (E): Promptly incision closure. (F): The schematic diagram of group allocation.

**Additional file 3：The biocompatible features of MenSCs and collagen gel**

(A): CCK8 analysis for MenSCs cultured with gel. (B): LDH analysis for the MenSCs cultured with gel. (C-F): The microstructure of collagen I scaffold with (C,E) or without (D,F) MenSCs under scanning electron microscopy.

**Additional file 4:** **Original western blot gels**

Original western blot gels of Fig. 1E for protein expression levels of TGF-β and IGF-I in different groups.
